# Heat Shock Proteins in Vascular Diabetic Complications: Review and Future Perspective

**DOI:** 10.3390/ijms18122709

**Published:** 2017-12-14

**Authors:** Stefania Bellini, Federica Barutta, Raffaella Mastrocola, Luigi Imperatore, Graziella Bruno, Gabriella Gruden

**Affiliations:** 1 Laboratory of Diabetic Nephropathy, Department of Medical Sciences, University of Turin, Corso Dogliotti 14, 10126 Turin, Italy; stefania.bellini@unito.it (S.B.); federica.barutta@unito.it (F.B.); limperat@unito.it (L.I.); graziella.bruno@unito.it (G.B.); 2 Department of Clinical and Biological Sciences, University of Turin, Corso Raffaello 30, 10125 Turin, Italy; raffaella.mastrocola@unito.it

**Keywords:** diabetes, diabetes complications, diabetic retinopathy, diabetic nephropathy, diabetic retinopathy, diabetic neuropathy, heat shock proteins, Heat shock protein (HSP) 27, HSP47, HSP60, HSP70, HSP90, biomarkers, albuminuria

## Abstract

Heat shock proteins (HSPs) are a large family of proteins highly conserved throughout evolution because of their unique cytoprotective properties. Besides assisting protein refolding and regulating proteostasis under stressful conditions, HSPs also play an important role in protecting cells from oxidative stress, inflammation, and apoptosis. Therefore, HSPs are crucial in counteracting the deleterious effects of hyperglycemia in target organs of diabetes vascular complications. Changes in HSP expression have been demonstrated in diabetic complications and functionally related to hyperglycemia-induced cell injury. Moreover, associations between diabetic complications and altered circulating levels of both HSPs and anti-HSPs have been shown in clinical studies. HSPs thus represent an exciting therapeutic opportunity and might also be valuable as clinical biomarkers. However, this field of research is still in its infancy and further studies in both experimental diabetes and humans are required to gain a full understanding of HSP relevance. In this review, we summarize current knowledge and discuss future perspective.

## 1. Introduction

Vascular complications are the major causes of morbidity and mortality in people with diabetes (DM). Although their underlying mechanisms are not fully understood, advanced glycated end products (AGEs), oxidative stress, and a low-grade inflammation are believed to play a key role in mediating hyperglycemia-induced cell dysfunction and damage [1]. There is thus the need to identify therapies either blocking cascades leading to cell injury or enhancing the effectiveness of endogenous cytoprotective machineries.

Heat shock proteins (HSPs) are a family of proteins highly conserved throughout evolution because of their key role in cytoprotection. Both pharmacological and genetic modulation of HSP expression has been tested in animal models as a strategy to enhance cytoprotection in the context of DM. Moreover, the discovery that HSPs can be released in the extracellular space has prompted clinical studies investigating the potential use of both HSPs and anti-HSP antibodies as serum biomarkers of DM complications.

In this review, we summarize current knowledge of the role of HSP27, HSP47, HSP60, HSP70, and HSP90 in DM complications and discuss the potential clinical relevance.

## 2. Diabetes Micro and Macrovascular Complications

People with DM are two to four times more likely to develop macrovascular diseases, such as coronary artery disease, stroke, and peripheral vascular disease. Moreover, cardiovascular events are the predominant cause of death in patients with DM [2].

Microvascular complications, affecting the eye, the kidney, and the nervous system, also have a significant impact. In the Western World diabetic nephropathy (DN) is a leading cause of end stage renal disease requiring renal replacement therapy. Additionally, patients with DN have an increased risk of cardiovascular morbidity and mortality. Hyperglycemia, hypertension, and a low-grade inflammation are the major determinants of the renal damage, comprising podocyte injury, mesangial matrix accumulation, tubule-interstitial fibrosis and resulting in albuminuria and progressive renal function decline [1,3].

Diabetic retinopathy (DR), the major cause of blindness in adults, is characterized by apoptosis of both pericytes and retinal endothelial cells, leading to disruption of the blood-retinal barrier and capillary loss. Vascular damage causes retinal ischemia and overexpression of angiogenic factors, which are responsible for the abnormal retinal neovascularization that characterizes the advanced proliferative phase of the complication [4].

DM also affects both the central and peripheral nervous system. Distal symmetric polyneuropathy (DSP), the most prevalent form of diabetic neuropathy, is due to axon degeneration secondary to both metabolic abnormalities and microvessel injury. DSP affects predominantly sensory neurons and causes paraesthesia, allodynia, hyperalgesia, and subsequently hypoalgesia [5]. In the central nervous system (CNS), DM induces a variety of functional alterations resulting in cognitive deficits that are collectively referred to as diabetic central neuropathy. Structurally, there is neuronal apoptosis and proliferation of both astrocytes and microglia in several brain areas and particularly in the hippocampus that is important in both learning and memory and highly susceptible to oxidative stress [6].

## 3. Heat Shock Proteins

Heat shock proteins (HSPs) are ubiquitous proteins classified in various families based on their molecular weight (HSP100, HSP90, HSP70, HSP60, HSP40, and small HSPs). Recently, a new classification has been proposed that divide HSPs in the following groups: HSPH (HSP110), HSPC (HSP90), HSPA (HSP70), DNAJ (HSP40), HSPB (small HSP) and the chaperonin families HSPD/E (HSP60/HSP10) and TRiC (also known as chaperonin-containing TCP-1 or CCT) [7] (Table 1).

HSPs comprise both constitutively expressed and inducible forms. Induction of HSPs occurs in response to stressors, such as thermal and osmotic stress, heavy metals, hypoxia, ischemia, and it is orchestrated by the transcription factor heat shock factor 1 (HSF1), which is the master regulator of the “stress response” [8].

As molecular chaperones, HSPs regulate the biosynthesis, folding/unfolding, transport, and assembly of cellular proteins and some HSPs also favour activation of associated proteins. Following cellular stress, induced HSPs facilitate refolding, prevent aggregation of misfolded proteins, and assist in the proteasomal degradation of irreversibly damaged proteins. Furthermore, HSPs inhibit apoptosis, protect against both oxidative stress and inflammation, and increase resistance to subsequent stresses (tolerance).

Although HSPs are predominantly intracellular cytoprotective machineries, they can also be exposed on the plasma membrane and released in the extracellular space, resulting in detectable levels of HSPs in the bloodstream. As HSPs do not possess signal sequences, they are not exported through the classical endoplasmic reticulum (ER)-Golgi complex secretory pathway and alternative mechanisms, including release within exosomes, are involved [9]. Extracellular HSPs have been implicated in cell-cell communication and both immune and inflammatory processes.

### 3.1. Heat Shock Protein 47—SerpinH1

Heat shock protein 47 (HSP47), a glycoprotein localized in the ER, is a collagen-specific chaperone. In particular, HSP47 inhibits collagen aggregation by binding to pro-collagen in the ER and facilitate triple helix formation. Gene ablation studies confirmed that HSP47 is essential for collagen maturation as HSP47-deficient cells contained misfolded pro-collagen aggregates in the ER that were then degraded by autophagy [10].

In the context of DM, most of the research has focused on the role of HSP47 in DN as excessive accumulation of extracellular matrix components, including collagen, is both a characteristic feature of DN and a key determinant of renal function loss. There is evidence of HSP47 overexpression in both human and experimental DN. A study performed on renal tissue from patients with DN has shown that overexpression of type III collagen in the interstitial space and of type IV collagen in the sclerotic glomeruli co-localized with an enhanced HSP47 expression by renal resident cells [11]. Similarly, in advanced experimental DN, expression of HSP47 was increased in mesangial cells, stressed podocytes, and tubular epithelial cells in parallel with increased both type III and type IV collagen deposition, glomerulosclerosis, and tubulointerstitial fibrosis [12]. Furthermore, HSP47 was induced during epithelial-mesenchymal transition, a key event underlying the development of renal fibrosis in chronic kidney diseases, including DN [13].

Formation of AGEs has been implicated in DM-induced HSP47 overexpression. In experimental diabetes, OPB-9195, an inhibitor of AGE formation, prevented not only transforming growth factor (TGF)-β1 and collagen expression, but also HSP47 induction [14]. Also, exposure of mesangial cells to AGEs induced HSP47 expression in a TGF-β1-dependent manner [14]. Although the intracellular signalling pathway whereby TGF-β1 induces HSP47 remains to be fully elucidated, in vitro experiments on proximal tubular cells suggest that activation of the signalling molecules extracellular signal-regulated kinase 1/2 (ERK1/2) and Jun kinase (JNK) is involved [15]. Moreover, in non-renal cells TGF-β1 can also enhance HSP47 expression by inhibiting microRNA-29b [16].

Inhibition of HSP47 has been proposed as a strategy for the management of fibrotic disorders and studies in a variety of murine models of fibrosis confirmed that knocking-down HSP47 decreased the accumulation of collagen and prevented fibrosis, as recently reviewed [17]. Although there are no data on HSP47 blockade/ablation in DN, studies performed in experimental both glomerulonephritis and tubulo-interstitial fibrosis have provided a proof of concept that HSP47 suppression can ameliorate renal fibrosis [18,19,20]. However, autophagy is impaired in DN and HSP47 deletion caused accumulation of pro-collagen aggregates leading to apoptosis in cells with altered autophagy [21]; therefore, the potential benefit of HSP47 blockade in DN must be confirmed. Of interest, a recent screening of compounds that inhibit the interaction between HSP47 and collagen has identified AK778 and its cleavage product Col003 as molecules that can inhibit collagen secretion [22] and these compounds might be exploited in the future for pharmacological intervention.

**Figure 1:**
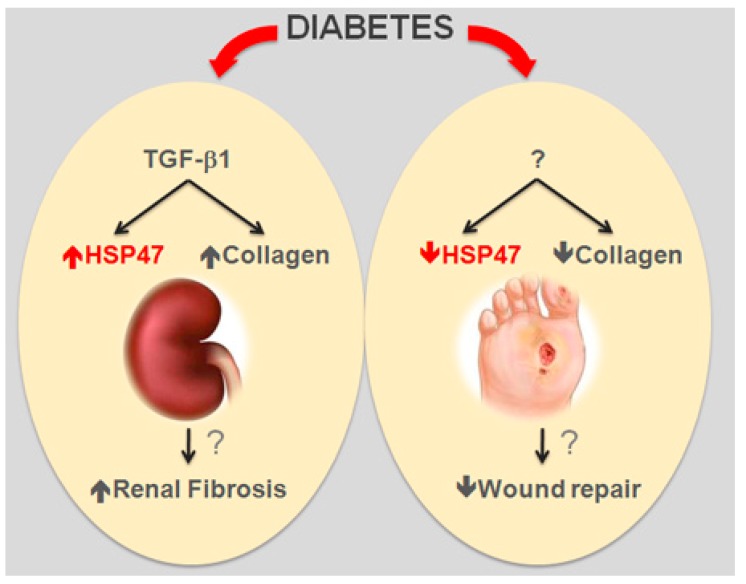
Role of HSP47 in Diabetic Complications. The collagen-specific chaperon HSP47 is overexpressed (up arrow) in experimental diabetic nephropathy and downregulated (down arrow) in diabetic wounds. This is paralleled by consensual changes in collagen expression and may be relevant in renal fibrosis and wound healing. TGF-β1: transforming growth factor-β1; ?: unproven hypothesis.

There are limited data on the potential role of HSP47 in other chronic complications of DM. However, collagen production is important in the healing process and HSP47 expression in wounds was reduced in both human and experimental diabetes [23,24], suggesting that insufficient HSP47 induction may interfere with wound healing in the context of DM. This implies that potential intervention strategies reducing HSP47 expression to protect the kidney may have the disadvantage to delay wound repair (Figure 1).

In summary, HSP47, which is important in collagen maturation, is upregulated in DN and downregulated in diabetic wounds with parallel changes in collagen levels. However, evidence of a beneficial effect of therapeutic strategies targeting HSP47 in these conditions is lacking.

### 3.2. Heat Shock Protein 27—HSPB1

HSPB1 (also known as HSP27 in both humans and rats and HSP25 in mice) belongs to the family of the small HSPs. Besides having a molecular chaperon function, HSPB1 stabilises the cytoskeleton through its actin-capping activity, prevents apoptosis, and reduces oxidative stress by increasing both glutathione levels and glyceraldehyde 3-phosphate dehydrogenase (GAPDH) activity. HSPB1 forms high molecular weight multimers and undergoes multiple phosphorylations predominantly through a p38 mitogen-activated protein kinase (p38MAPK)/MAPK-activated protein 2 (MK2) pathway. In vitro, phosphorylation leads to disaggregation of HSPB1 multimers, possibly affecting HSPB1 both cellular localization and function. Moreover, phosphorylation favours actin polymerization by inhibiting HSPB1 actin-capping activity [25]. Finally, HSPB1 can also be released into the extracellular space through both lysoendosomal and exosomal pathways [26].

#### 3.2.1. Diabetic Nephropathy

Podocytes are important constituent of the glomerular filtration barrier and the slit diaphragm, a junction connecting the foot processes of neighbouring podocytes, is the major restriction site to glomerular protein filtration. DM alters podocyte both function and structure and this eventually leads to podocyte foot processes effacement, apoptosis, and proteinuria [1]. Given the important role of HSPB1 in both inhibiting apoptosis and controlling cytoskeleton dynamics, HSPB1 is expected to be relevant in podocyte pathophysiology. Consistent with this notion, HSPB1 is highly expressed by podocytes in the normal kidney [27]. Moreover, in experimental DM there was an increased glomerular expression of either total or phosphorylated HSPB1 [27,28,29,30], particularly in podocytes, and a transition from large to small HSPB1 oligomers in isolated diabetic glomeruli [31]. Moreover, exposure of cultured podocytes to high glucose, angiotensin II, and mechanical stretch to mimic glomerular hypertension enhanced HSPB1 either expression [28] or phosphorylation [27] and silencing of HSPB1 exacerbated high-glucose-induced podocyte apoptosis [28].

Likely, HSPB1 phosphorylation/induction is a cytoprotective mechanism aimed to limit both oxidative stress and apoptosis. However, HSPB1 phosphorylation causes actin uncapping and may reduce the stability of the podocyte cytoskeleton that is crucial in podocyte function. In keeping with this hypothesis an increase in both total and phosphorylated HSPB1 was reported in puromycin-induced nephrotic syndrome [32]. Deletion of MK2, which phosphorylates HSPB1, was not beneficial in an animal model of streptozotocin (STZ)-induced diabetes, failing to show a functional relevance of the p38/MK2/HSPB1 pathway in DN [29]. However, HSPB1 phosphorylation was not entirely abrogated in these mice because other kinases can phosphorylate HSPB1 beside MK2; therefore, strategies that completely block phosphorylation are required to clarify the potential role of HSPB1 in the pathogenesis of the diabetic proteinuria.

There are no data on circulating levels of HSPB1 in DN except for a small study that reported a rise in plasma HSPB1 levels in patients with type 2 DM (DM2) and DN compared with DM2 subjects without any chronic complications [33].

#### 3.2.2. Diabetic Retinopathy

Both expression profiling and next generation sequencing analyses of the retina from STZ-induced diabetic mice showed that HSPB1 is overexpressed in early as well as advanced DR [34,35,36]. This is considered a compensatory response to counteract DM-induced both oxidative stress and apoptosis [37]. Although data on the expression of HSPB1 protein are more conflicting with some studies reporting enhanced [38,39] and others reduced [40,41] HSPB1 expression, the phosphorylated form of HSPB1 was increased in the diabetic retina and inhibition of HSPB1 phosphorylation using a p38 inhibitor ameliorated the retinal vascular injury in vivo [38]. DM also affects the neural components of the retina and induces apoptosis of retinal ganglion cells (RGC) that are of critical importance to the visual system. HSPB1 is not constitutively expressed by RGC, but can be induced by stresses [42] and we found that in RGC from diabetic mice there was an overexpression of both HSPB1 and markers of oxidative stress and apoptosis [39].

Recently, HSPB1 has also been implicated in the neovascularization of the retina. This is based predominantly on in vitro studies on endothelial cells, highlighting a complex interaction between HSPB1 and vascular endothelial growth factor (VEGF), the major inducer of retinal neovascularization in DR. VEGF promoted HSPB1 phosphorylation via a stress-activated protein kinase 2 (SAPK-2)/p38 pathway, thus favouring both cytoskeleton reorganization and endothelial cell migration, possibly supporting angiogenic sprouting [43]. Moreover, HSPB1 phosphorylation reduced the release of HSPB1 in the extracellular space, where extracellular HSPB1 (eHSPB1) binds to and blocks VEGF [44], but also enhanced intracellular VEGF expression by engaging the Toll-Like Receptor 3 (TLR3) on endothelial cells [45]. Although the relevance of these findings to proliferative DR remains to be proven, they underscore the importance of both intracellular and extracellular HSPB1 in fine-tuning VEGF pro-angiogenic activity.

#### 3.2.3. Diabetic Neuropathy

HSPB1 is considered highly relevant in neurons both function and protection. Accordingly, HSPB1 mutations can cause hereditary polyneuropathies [46,47] and induction of HSPB1 is crucial in both axonal regeneration and neuron survival in experimental models of axon injury [48,49].

Both atrophic and degenerative changes of dorsal root ganglia (DRG) neurons have been described in animal models of DM, though DRG neurons are relatively preserved compared to those in animal models of axotomy. Data on HSPB1 expression in DRG neurons are conflicting. A study did not find any change, while HSPB1 was the only marker of plasticity/stress to exhibit enhanced gene expression in another report [50,51]. However, there is evidence of a beneficial effect of HSPB1 in DSP as HSPB1 overexpression in neurons of diabetic mice conferred protection against a range of neuropathic abnormalities, including loss of footpad thermal sensation, mechanical allodynia, loss of epidermal innervation, reduced sensory conduction velocity [52]. In DM, changes of HSPB1 expression have also been shown in the CNS. Indeed, HSPB1 was overexpressed in the hippocampus of STZ-induced diabetic mice, predominantly in CA1 neurons and activated glial cells, and this was paralleled by astrogliosis and overexpression of oxidative stress markers [53].

Circulating levels of HSPB1 have also been proposed as a biomarker of DSP. In the large cohort of patients with type 1 DM (DM1) of the EURODIAB PCS study, we found that higher circulating HSPB1 levels conferred a twofold increased risk of DSP, independently of known risk factors and confounders [54]. Although the mechanism of the rise of serum HSPB1 levels in patients with DSP is unclear, this finding is of relevance as the lack of circulating markers for DSP represents an important limit of clinical research in the field. This association appears specific of DSP in DM1, as no association was found with autonomic neuropathy [55] and even an inverse association reported in DM2 [56]. However, prospective studies exploring the potential relevance of HSPB1 as a positive predictor of DSP have not yet been performed.

#### 3.2.4. Diabetic Macrovascular Diseases

HSPB1 is considered of relevance in vascular homeostasis because of its anti-apoptotic and chaperon properties. Moreover, HSPB1 also controls the actin cytoskeleton, suggesting a potential involvement in vascular remodelling. In human atherosclerotic plaques, there is a reduction in both total and phosphorylated HSPB1 expression [57,58,59,60]. HSPB1 downregulation is even greater in complicated lesions, raising the possibility that HSPB1 deficiency plays a role in atherosclerosis progression. In keeping with this, in high fat diet-fed (HFD)-ApoE^−/−^ mice, HSPB1 overexpression ameliorated the phenotype of atherosclerotic plaques, reducing lesion area, cholesterol content, apoptosis, and macrophage accrual, while increasing intimal both vascular smooth muscle cells (VSMC) and collagen content [61]. Although there are no available data in diabetic animals, inhibition of HSPB1 phosphorylation enhanced apoptosis in endothelial cells exposed to high glucose [62]. Moreover, a reduction in GAPDH activity is considered a converging point of multiple deleterious pathways activated by hyperglycemia and a key step in the pathogenesis of DM complications [63]; therefore, the ability of HSPB1 to activate GAPDH may be particularly advantageous in DM.

HSPB1 is also released in the extracellular space and recent studies suggest that eHSPB1 plays an important role in atherosclerosis. HSPB1 secretion was drastically reduced in atherosclerotic lesions and almost abolished in complicated plaques [58]. Extracellular HSPB1 appears to diminish the lipid engulfment of monocyte/macrophages by both reducing the uptake of atherogenic lipoproteins and enhancing cholesterol efflux. Indeed, eHSPB1 was shown to block and downregulate the macrophage scavenger receptor A [64,65]. Moreover, binding of eHSPB1 to the macrophage TLR4 induced the nuclear factor kappa-light-chain-enhancer of activated B cells (NF-κB)-mediated release of the granulocyte-macrophage colony-stimulating factor (GM-CSF), which in turn promoted cholesterol efflux by enhancing ATP-binding cassette (ABC) transporter activity [66]. Accordingly, in vivo in ApoE^−/−^ mice, HSPB1 overexpression significantly decreased cholesterol content in the plaque area via a GM-CSF-dependent mechanism [66]. On the other hand, eHSPB1 may also have deleterious effects as binding of eHSPB1 to endothelial TLR2-3-4 activates NF-κB [45,67]. Moreover, the release of phosphorylated HSPB1 from platelets was correlated to accelerated platelet aggregation in patients with DM2 [68,69].

Clinical studies have explored if changes in circulating HSPB1 occur in a mixed population of non-diabetic and DM2 patients with CVD. Serum HSPB1 levels were increased in patients with acute coronary syndrome [60] and ischemic stroke [70], likely because of cell injury. On the contrary, they were reduced in patients with stable CVD and predicted subsequent major clinical events in patients with established CAD, but not in a primary prevention cohort [58,71,72,73]. At variance, we did not observe any association between HSPB1 and CVD in patients with DM1 [54]. Although the presence of HSPB1 autoantibodies in the serum may represent a potential confounder by shielding HSPB1, there was no difference in anti-HSPB1 levels between DM1 patients with and without vascular complications [74].

**Figure 2:**
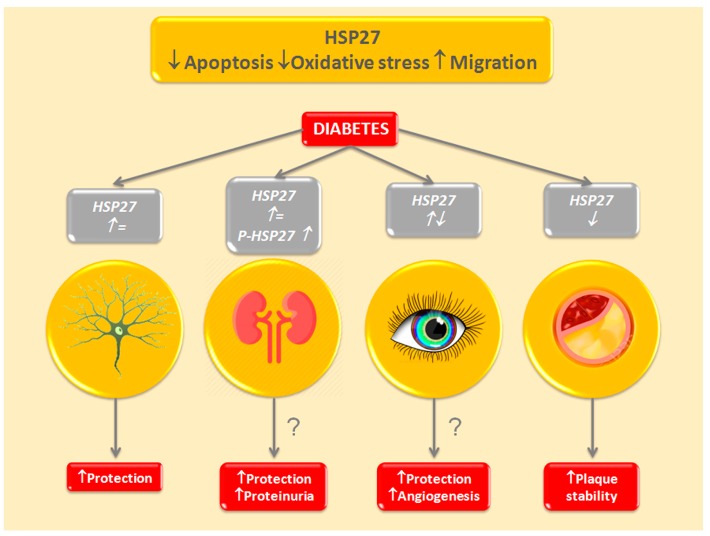
HSPB1/HSP27 in Diabetic Complications. HSPB1/HSP27 has anti-apoptotic and anti-oxidative effects and controls cell migration acting on the actin cytoskeleton. Expression of total and/or phosphorylated HSPB1/HSP27 (P-HSPB1/HSP27) is altered (up arrow: overexpressed; down arrow: downregulated) in target organs of diabetic complications (neurons, kidney, retina, vessel wall). HSPB1/HSP27 has neuroprotective effects and stabilizes atherosclerotic plaques. The role of HSPB1/HSP27 in diabetic nephropathy and retinopathy is undetermined; however, HSPB1/HSP27 is considered cytoprotective, while P-HSPB1/HSP27 might have deleterious effects (proteinuria, retinal neovascularization). ?: unproven hypothesis.

In summary, levels of HSPB1/phosphorylated HSPB1 are either unchanged or enhanced in diabetes microvascular complications. On the contrary, both total and phosphorylated HSPB1 are downregulated in atherosclerotic plaques and even more in the unstable ones. There is evidence of a beneficial effect of HSPB1 overexpression in animal models of atherosclerosis and in diabetic animals with DSP. Circulating levels of HSPB1 have been proposed as a biomarker of DSP (Figure 2).

### 3.3. Heat Shock Protein C—HSP90

The highly conserved HSPC/HSP90 family comprises 5 members HSPC1/HSP90AA1 (cytosol inducible), HSPC2/HSPAA2 (cytosol inducible), HSPC3/HSPAB1 (cytosol constitutive), HSPC4/GRP94 (ER), and HSPC5/TRAP1 (mitochondria), but most of the studies in DM complications were performed on the cytosolic members of this family.

The biological activity of HSPC as chaperon depends on its capacity to bind and hydrolyse ATP, which drives the closed conformation of active HSPC. Besides its chaperon function, HSPC facilitates the maturation of hundreds of proteins, also named “clients”, that are involved in a variety of cellular processes, including cell survival, signal transduction, transcriptional regulation, and inflammation. A large number of co-chaperones, including HSPA/HSP70, interact with HSPC and regulates its conformational changes during client processing. The transcription factor HSF1 is not only an inducer, but also a client of HSPC. In fact, the HSPC-HSPA complex binds to HSF1 and keeps it in an inactive state. If chaperones are required elsewhere and no longer available for HSF1 inhibition, free HSF1 moves to the nuclei with subsequent increase of heat shock gene transcription. Therefore, HSPC links the stress status of cells to HSP gene expression [75].

As HSPC can promote cell survival, migration, inflammation, and angiogenesis, it is considered a very promising target in cancer therapy. This has led to development of specific HSPC inhibitors and today more than a dozen of HSPC inhibitors are undergoing clinical testing in humans as chemotherapeutics [76]. Recently, these compounds have also been tested in diabetic animals and these studies have proven the benefit of HSPC blockade in diabetic complications.

#### 3.3.1. Diabetic Nephropathy

In the normal kidney, HSPC is highly expressed in the outer medulla where it interacts with the steroid hormone receptor. Within the glomeruli HSPC is present predominantly in mesangial cells and podocytes. We did not observe any change in HSPC expression in either the kidney of DM1 animals or glomerular cells exposed in vitro to high glucose and mechanical stretch [27] and another group has even reported a HSPC downregulation in the renal cortex of DM2 mice [77]. Despite the lack of HSPC overexpression, intervention studies have shown that HSPC inhibition is beneficial in DN. In db/db mice fed with a HFD, the HSPC inhibitor 17-dimethylaminoethylamino-17-demethoxygeldanamycin (17-DMAG) preserved kidney function, ameliorated both glomerular and tubular damage, and improved survival [78]. Because HSPC also modulate lipid homeostasis [79], amelioration of lipid profile may partially explain the renal benefit in this model of combined hyperglycemia and dyslipidemia. However, a more recent study in STZ-induced diabetic ApoE^−/−^ mice has shown that 17-DMAG can reduce albuminuria, mesangial expansion, and inflammation independently of both hyperglycemia and dyslipidemia [80]. This benefit may be due to reduced inflammation as HSPC inhibition diminished both NF-κB and the activation of signal transducer and activator of transcription (STAT), and HSPC is known to stabilise two kinases (IKK and JAK2) that activate the NF-κB and STAT pathways. Alternatively, the renal protective effect may be secondary to HSF-1-mediated HSPA/HSP70 induction [80]. Furthermore, an inhibitory effect on the TGF-β1 pathway cannot be excluded based on recent studies showing an anti-fibrotic effect of HSPC blockade [81,82,83].

#### 3.3.2. Diabetic Retinopathy

HSPC is highly expressed in the rat retina [84] and undergoes downregulation in OLETF rats, a model of DM2 [85]. This downregulation is believed to contribute to RGC apoptosis, as HSPC is important in stabilising the phosphorylated form of the signalling molecule Akt that plays a key role in RGC survival. On the other hand, HSPC may also be deleterious in DR because HSPC stabilises the transcription factor hypoxia-inducible factor (HIF)-1 that induces the expression of pro-angiogenic factors involved in the development of proliferative DR. Consistent with this, the HSPC inhibitor geldanamycin and its derivative 17-*N*-allylamino-17-demethoxygeldanamycin (17-AAG) abolished the release of the angiogenic factors VEGF, integrin-linked kinase (ILK), and stromal cell-derived factor (SDF)-1 in cultured retinal pigment epithelial cells [86,87]. In addition, two novel HSPC blockers, the deguelin analogues SH-1242 and SH-1280, reduced both HIF-1 half-life and VEGF upregulation in vivo and suppressed hypoxia-mediated retinal neovascularization [88]. These compounds also inhibited vascular leakage in a model of STZ-induced DM, suggesting a potential benefit in DM-induced macular oedema [88]. Therefore, potential therapeutic strategies targeting HSPC in DR should be fine-tuned in order to antagonize hypoxia-induced angiogenesis without enhancing apoptosis.

#### 3.3.3. Diabetic Neuropathy

HSPC inhibition has been used as a strategy to increase the expression of neuroprotective HSPA/HSP70 [89]. In STZ-induced diabetic mice, treatment with the HSPC inhibitor KU-32 reversed both sensory hypoalgesia and altered nerve conduction velocity (NCV). This occurred through induction of HSPA/HSP70 as no benefit was observed in HSPA/HSP70 knockout animals [90]. In most diabetic animals, reversal of these functional deficits was associated with an increased density of intra-epidermal nerve fibre bundles [91], indicating that the clinical benefit of HSPC inhibition was mainly due to fibre regeneration. In addition, in vitro KU-32 prevented high glucose-induced death of DRG neurons and decreased neuregulin-1 (NRG-1)-induced demyelination [90]. A recent study has clarified the underlying mechanism by showing that KU-32-induced HSPA/HSP70 upregulation causes proteosomal degradation of c-Jun, a downstream signalling molecule of NRG-1 [92]. Improvement of mitochondrial bioenergetics has recently emerged as another mechanism of the neuroprotective effects of KU-32. In vitro, KU-32 decreased hyperglycemia-induced oxidative stress and improved mitochondrial bioenergetics in sensory neurons [93]. Moreover, ex vivo maximal respiratory capacity was reduced in sensory neurons isolated from STZ-induced diabetic animals and HSPC inhibition ameliorated this abnormality in vivo [91] in an HSPA/HSP70-dependent manner [94].

#### 3.3.4. Diabetes Macrovascular Complications

A decline in vascular bioavailability of nitric oxide (NO) is a key determinant of endothelial dysfunction in DM [95]. The formation of a complex between HSPC and endothelial NO synthase (eNOS) is a prerequisite for subsequent Akt-mediated eNOS activation resulting in NO generation. Therefore, several studies explored whether formation of HSPC-eNOS complexes is altered in DM. HSPC-eNOS complexes were reduced in the aortic endothelium of diabetic rats [96]. Moreover, in vitro exposure of endothelial cells to high glucose reduced formation of HSPC-eNOS complexes because high glucose decreased HSPC expression, promoted HSPC translocation to the cell surface, and enhanced HSPC interaction with alternative clients as IKKβ [96,97,98]. In contrast, metformin enhanced HSPC-eNOS interaction by inducing eNOS phosphorylation [99]. Therefore, DM can impair NO formation at least in part by reducing the stabilising effect of HSPC on eNOS.

However, studies in both human and experimental atherosclerosis suggest that HSPC may be deleterious and that HSPC inhibition may represent a potential therapeutic strategy in macrovascular diseases. In human atherosclerotic lesions, there was a HSPC upregulation [100], particularly in the unstable shoulder region of the plaque, while stable plaques were enriched in HSPA/HSP70 [101]. In addition, serum HSPC levels were higher in patients with atherosclerosis and antibodies against HSPC were detected exclusively in atherosclerotic patients and not in healthy controls [100]. Treatment of STZ-induced diabetic ApoE^−/−^ mice with the HSPC inhibitor 17-DMAG significantly reduced atherosclerotic lesions and induced a more stable plaque phenotype [80]. Similarly, HSPC inhibition diminished both lesion size and inflammation in STZ-induced diabetic animals [102]. These atheroprotective effects of HSPC inhibition were likely due to HSPA/HSP70 induction and/or inhibition of transcription factors affecting both inflammation and oxidative stress (nuclear factor erythroid-derived like 2 or Nrf2, NF-κB, STAT) [80,102].

On the other hand, HSPC and in particular HSP90α can be beneficial in wound healing. Topical application of a fragment of secreted HSP90α (F-5) accelerated diabetic wound healing in mice and showed greater efficacy compared to other growth factors because it enhanced both epidermal and dermal cell recruitment overriding the inhibitory effects of hyperglycemia and TGF-β1 on cell migration [103].

In conclusion, HSPC has both beneficial and deleterious effects in the context of DM macrovascular diseases. Despite the availability of specific HSPC inhibitors, it is thus difficult to pharmacologically reduce HSPC activity without causing relevant unwanted side effects.

In summary, levels of HSPC are either unchanged or downregulated in microvascular complications, while an HSPC overexpression is observed in stable regions of atherosclerotic plaques. HSPC inhibition was shown to be beneficial in all micro and macrovascular diabetes complications with the exception of diabetic wounds where a benefit of treatment with HSP90α was proven.

### 3.4. Heat Shock Protein A—HSP70

In humans, constitutive HSC70/HSP73 and inducible HSP72/iHSP70 are the major members of the HSPA/HSP70 family. HSPA/HSP70 favours protein (re)-folding, transports proteins through membranes to enable their delivery to organelles, it recruits proteins to the proteasome for turnover, and brings proteins to the endosome/lysosome compartment for autophagy. These diverse functions are accomplished by interactions with a variety of partners, including HSPC/HSP90, J proteins, negative regulatory factors (NEFs), lysosome-associated membrane protein 2A (LAMP-2A), and even lipids. In addition, HSPA/HSP70 blocks multiple apoptotic pathways and inhibits inflammation by sequestering NF-κB. Moreover, HSPA/HSP70 is induced by oxidative stress and prevents NF-κB-induced iNOS expression thereby limiting generation of both reactive oxygen species (ROS) and peroxynitrite. More recently, accumulating evidence indicates a role of extracellular HSPA/HSP70 as a modulator of inflammatory and immune processes [104].

#### 3.4.1. Diabetic Nephropathy

In the normal kidney, HSPA/HSP70 expression increases progressively along the cortico-papillary axis in parallel with the osmotic gradient [105]. In STZ-induced diabetic rats, HSPA/HSP70 was upregulated in the outer medulla, while glomerular expression was only modesty increased and exclusively in early DN [27]. Data on HSPA/HSP70 expression in cortical tubules are conflicting [27,77,106,107,108,109,110]; however, a recent study on kidney biopsies from patients with DN has shown an upregulation of HSPA/HSP70 in the cortical tubules [111]. Moreover, in vitro HSPA/HSP70 was overexpressed in both tubular cells exposed to high glucose and renal interstitial fibroblasts exposed to AGEs [107,108,110].

As described above, HSPA/HSP70 induction using HSPC inhibitors was beneficial in experimental models of early DN; however, recent evidence suggests that extracellular HSPA/HSP70 may favour DN progression by promoting tubule-interstitial inflammation. In vitro in proximal tubule cells, albumin strongly induced the release of eHSPA/eHSP70 that, in turn, triggered the overexpression of the inflammatory cytokines monocyte chemoattractant proteins 1 (MCP-1), tumor necrosis factor alpha (TNF-α), and macrophage inflammatory protein 2 (MIP2) via a TLR4-NF-κB pathway [111]. Likewise, in vivo in STZ-induced diabetic animals both TLR4 and HSPA/HSP70 were overexpressed in the tubules and either TLR4 deletion or treatment with a variety of HSPA/HSP70 inhibitors, acting on either intra or extracellular HSPA/HSP70, reduced albuminuria and markers of both inflammation and tubular injury [111]. Therefore, HSPA/HSP70 may be beneficial in early DN because of its intracellular cytoprotective activity, but when albuminuria develops extracellular HSPA/HSP70 may contribute to the tubular-interstitial damage that is a key driver of DN progression. Consistent with this, a small study performed in patients with DM2 showed an association between urinary HSPA/HSP70 levels and albuminuria [112]. Moreover, serum HSPA/HSP70 levels were higher in DM2 patients with albuminuria [113] and polymorphisms of the HSPA/HSP70 gene were associated with DN in DM2 patients [114].

#### 3.4.2. Diabetic Retinopathy

Expression of HSPA/HSP70 is globally enhanced in both diabetic retinas and retinal endothelial cells exposed to high glucose. There are also changes in HSPA/HSP70 distribution as HSPA/HSP70 was increased in the cytosol and reduced in the mitochondria [115]. Formation of complexes between HSPA/HSP70 and the matrix metallopeptidase 9 (MMP-9) was enhanced in murine diabetic retinas and this is of relevance because oxidative stress-induced MMP-9 activation plays a key role in DM-induced mitochondrial abnormalities leading to retinal endothelial cell apoptosis. Indeed, knocking-down MMP-9 in either diabetic animals or retinal endothelial cells exposed to high glucose reversed apoptosis, mitochondrial alterations, and abnormalities in HSPA/HSP70 both expression and distribution. The specific role of HSPA/HSP70 in this process has been only partially clarified; however, HSPA/HSP70 may be important in chaperoning MMP-9 to the mitochondria and thus in enhancing MMP-9-induced mitochondrial damage [115].

Data on the circulating levels of both HSPA/HSP70 and anti-HSPA/HSP70 antibodies in DR are scarce. However, a study in patients with DM2 reported a rise in serum HSPA/HSP70 levels in patients with DR (*n* = 50) compared with subjects without DR (*n* = 50) [116], while anti-HSPA/HSP70 levels were negatively and independently associated with DR in the large cohort of patients with DM1 in the EURODIAB PCS [117].

#### 3.4.3. Diabetic Neuropathy

HSPA/HSP70 has neuroprotective effects in a variety of neurological conditions. In the context of DM, several studies have shown that HSPA/HSP70 increases the ability of both neurons and Schwann cells to tolerate on-going diabetic insults by improving oxidative stress, mitochondrial function, and possibly inflammation, as recently reviewed [118]. In cultured sensory neurons, induction of HSPA/HSP70 increased the expression of the anti-oxidant enzyme superoxide dismutase 2 (SOD2). In diabetic animals, HSPA/HSP70 induction ameliorated DSP and abolished DRG overexpression of two pro-oxidant proteins: the enzyme NADPH oxidase 2 (NOX2) and thioredoxin interacting protein [93,94] that enhances oxidative stress by inhibiting anti-oxidant thioredoxin. HSPA/HSP70 is also believed to improve mitochondrial bioenergetics. Although pharmacological induction of HSPA/HSP70 does not modify mitochondrial protein expression, most mitochondrial proteins are imported to the mitochondria after transcription and the key role of HSPA/HSP70 in this transfer may explain its beneficial effect [94]. Finally, HSPA/HSP70 has an anti-inflammatory activity in neurons, predominantly due to a reduction in NF-κB activity. Intervention studies proving the relevance of HSPA/HSP70 in DSP have been performed using HSPC inhibitors to induce HSPA/HSP70 expression and they have been reported above.

#### 3.4.4. Diabetic Macrovascular Diseases

HSPA/HSP70 expression is enhanced in human atherosclerotic lesions. Moreover, in vitro studies have shown that oxidized LDLs can induce HSPA/HSP70 expression in activated macrophages, endothelial cells, and vascular smooth muscle cells. As HSPA/HSP70 is cytoprotective, this overexpression may be a reactive response aimed to limit atherogenic stressors-induced damage [119]. There is relatively little information on HSPA/HSP70 in diabetic macrovascular diseases; however, a recent study has shown that HSPA/HSP70 induction can ameliorate vascular dysfunction secondary to insulin-resistance [120]. Moreover, a HSPA/HSP70 polymorphism was associated in patients with DM2 with both cerebral ischemic events and the presence of unstable plaques [121].

HSPA/HSP70 can also be exposed on the plasma membrane in stressed cells and/or released into the extracellular space and subsequently into the bloodstream either through passive secretion or as a result of necrotic cell death [119,122]. The observation that serum HSPA/HSP70 levels are enhanced in patients with DM and correlate with C reactive protein (CRP) levels suggests that diabetes-related insults can enhance exposure/release of HSPA/HSP70 [123]. Extracellular HSPA/HSP70 has important modulatory effects on both innate and adaptive immunity, as recently reviewed [104], acting either as a cross-presenter of immunogenic peptides via major histocompatibility complex (MHC) antigens or as a chaperokine. Although eHSPA/eHSP70 appears to enhance both inflammatory and immune responses, recent studies suggest that eHSPA/eHSP70 can also have important anti-inflammatory effects, as recently reviewed [124] by activating both immunosuppressive regulatory T cells (Treg) and sialic acid-binding immunoglobulin superfamily lectin (SIGLEC) receptors that block inflammatory processes through direct interaction with the TLRs [125]. Clinical studies in the general population have reported an inverse association between levels of HSPA/HSP70 and anti-HSPA/HSP70 and both the presence and the severity of cardiovascular diseases (CVD) [126,127,128,129]. Furthermore, lower HSPA/HSP70 levels were a predictor of future development of atherosclerosis in subjects with established hypertension [130]. Similarly, our study on patients with DM1 from the EURODIAB PCS showed that serum anti-HSP70 antibody levels in the upper quartiles were associated with an almost 50% lower likelihood of micro- and macrovascular complications with respect to lower values, independently of conventional risk factors and markers of both inflammation and endothelial dysfunction. Subgroup analysis revealed a significant association with both DR and CVD [117]. Therefore, clinical data in subjects with and without DM suggest that HSPA/HSP70 and anti- HSPA/HSP70 are protective markers in CVD.

In summary, HSPA/HSP70 expression is either unchanged or more often increased in diabetic complications, including macrovascular diseases. HSPA induction using a HSPC inhibitor has beneficial effects in both diabetic nephropathy and neuropathy. However, eHSPA/eHSP70 can promote DN progression by enhancing tubule-interstitium inflammation. Circulating levels of anti-HSPA/HSP70 are a protective marker in CVD.

### 3.5. Heat Shock Protein D1—HSP60

HSPD1/HSP60 is a type I chaperonin and together with its co-chaperone HSPE1/HSP10 forms a chaperone system specific of the mitochondria. HSPD1/HSP60 has a central hydrophobic cavity that provides an isolated environment for both de novo protein folding and matrix protein refolding, and its chaperone activity is ATP-dependent. HSPD1/HSP60 is essential for the maintenance of mitochondrial both activity and biogenesis and undergoes upregulation in response to mitochondrial impairment, being thus considered a marker of mitochondrial stress [131]. Under stress conditions, HSPD1/HSP60 is also found in the cytosol, on the cell surface acting as a signal molecule for the immune system, and in the extracellular space where it can regulate both inflammatory and immune processes.

#### 3.5.1. Microvascular Complications

In the normal kidney, HSPD1/HSP60 content is greater in renal cortex and outer medulla with a distribution that mirrors the abundance of mitochondria [105]. Renal HSPD1/HSP60 expression was increased in STZ-induced diabetic rats, particularly in the outer medulla, whereas no changes were found in both the glomeruli and glomerular cells exposed to high glucose [27]. At variance with these results, a proteomic analysis performed on renal tissue from STZ-induced diabetic rats reported a marked HSPD1/HSP60 downregulation [132], though renal distribution was not described. Recently, a global network investigation of previous proteomic analyses in DN has identified HSPD1/HSP60 as a central node in protein-to-protein interaction. Functional validation performed by studying tubular cells exposed in vitro to high glucose has shown that HSPD1/HSP60 silencing can modestly diminish high glucose-induced cell dysfunction [133].

Few studies assessed HSPD1/HSP60 in other microvascular complications. In the diabetic retina, HSPD1/HSP60 expression was globally enhanced, but reduced in the mitochondria, suggesting DM-induced mitochondrial stress. Moreover, in retinal endothelial cells, increased mitochondrial MMP-2 activity was shown to alter mitochondrial integrity by modulating both HSPD1/HSP60 and connexin-43 and allowing cytochrome c to leak out with activation of the apoptotic machinery [134].

Finally, HSPD1/HSP60 was overexpressed in the cell bodies of CA1 and CA3 hippocampal neurons of STZ-induced diabetic rats together with enhanced hippocampal levels of manganese superoxide dismutase enzyme (MnSOD) [135], suggesting that oxidative stress may induce a compensatory HSPD1/HSP60 overexpression in the CNS.

#### 3.5.2. Macrovascular Complications

In both humans and murine models of atherosclerosis, there is evidence of HSPD1/HSP60 overexpression in atherosclerotic lesions [136,137,138], peaking in early and decreasing in advanced lesions [136].

Moreover, exposure of endothelial cells to various stresses, including DM-associated stresses, induced HSPD1/HSP60 both expression on the cell surface and extracellular release, as recently reviewed [138]. Extracellular HSPD1/HSP60 can have direct auto/paracrine pro-inflammatory effects. For instance, exposure of VSMCs to platelet-derived growth factor BB (PDGF-BB) enhanced the release of HSPD1/HSP60 that, in turn, induced VSMCs migration via a TLR4-ERK-dependent mechanism [139]. Furthermore, HSPD1/HSP60 exposure/release can also elicit an immune/autoimmune reaction. All subjects acquire adaptive immunity against bacterial HSP60/65 by infection and, because there is a high degree of homology between mammalian and bacterial HSPD1/HSP60, this can trigger an immune cross-reaction against human HSPD1/HSP60. Furthermore, chemically altered human HSPD1/HSP60, derived from either damaged or necrotic cells, can also induce an autoimmune reaction against human HSPD1/HSP60 [138,140]. Studies performed in animal models of atherosclerosis suggest that this immune/autoimmune reaction can contribute to atherogenesis. T-cell clones recognizing HSPD1/HSP60 were found in atherosclerotic plaques and immunization with HSP60/65 increased atherosclerosis in animals [141,142,143]. Moreover, in healthy subjects the prevalence of early atherosclerotic lesions was associated with anti-HSPD1/HSP60 reactivity of peripheral T cells [144,145], and both cross-sectional and prospective studies showed a direct association between anti-HSP60/65 levels and CVD [138,146,147,148]. Moreover, HSPD1/HSP60 autoantibodies, isolated from patients with CVD, could promote atherosclerosis in ApoE^−/−^ mice. Of interest, this effect was prevented by mice pre-immunization with the F(ab)2 segments of the human-derived HSPD1/HSP60 antibody [149], raising the possibility that tolerizing vaccines may represent a therapeutic strategy in atherosclerosis [150].

By comparison there is relatively little information on HSPD1/HSP60 in patients with DM and CVD. However, a cross-sectional study performed on 855 patients with DM2 showed that the percentage of subjects with detectable circulating levels of HSPD1/HSP60 was greater in patients with CVD as compared to subjects without CVD and an history of myocardial infarction was associated with higher levels of HSPD1/HSP60 [151]. Another study reported that patients with both DM2 and peripheral vascular disease had anti-HSP60/65 levels comparable to healthy controls [152]. Similarly, we did not observe any change in circulating anti-HSPD1/HSP60 levels in DM1 patients with and without CVD [117].

In summary, HSPD1/HSP60 is globally enhanced in diabetic complications, though a reduction of mitochondrial HSPD1/HSP60 has been reported in diabetic retinopathy, suggesting mitochondrial stress resulting in translocation of HSPD1/HSP60 from the mitochondria to the cell membrane/extracellular space where HSPD1/HSP60 can elicit an immune/autoimmune reaction contributing to cell damage.

## 4. Summary of Evidence

**Figure 3:**
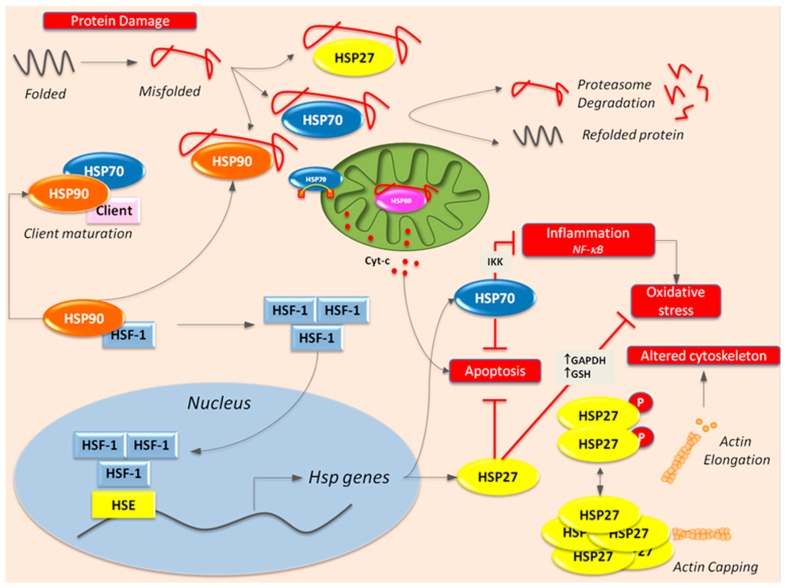
Cytoprotective Effects of HSPs in Diabetes. HSP90 binds to the transcription factor HSF1 and keeps it in an inactive state. If HSP90 is required elsewhere, free HSF1 moves to the nuclei and induces HSP transcription. HSP90 facilitates the maturation of several proteins, known as HSP90 “clients”. HSPB1/HSP27 forms high molecular weight multimers that stabilize the cytoskeleton, but the phosphorylated form loses its actin-capping activity. HSP60 is located in the mitochondria and HSP70 shuttle cytosolic protein to the mitochondria. Diabetes causes cell damage by inducing oxidative stress, inflammation, protein damage, cytoskeleton remodeling, and apoptosis (red squares). Diabetes-induced misfolded proteins either are rescue by HSPs or undergo proteasome degradation. Besides favoring protein refolding, HSPB1/HSP27 prevents apoptosis and reduces oxidative stress (red T symbols) by increasing both glutathione (GSH) levels and glyceraldehyde 3-phosphate dehydrogenase (GAPDH) activity. HSP70 inhibits apoptosis and reduces NF-κB-dependent inflammation (red T symbols) with consequent reduction of oxidative stress.

Given their key role in cytoprotection, HSPs can theoretically counteract the deleterious effects of diabetes in target cells of diabetes chronic complications (Figure 3). Overall data reported above show that expression of HSPs is modulated in diabetic complications with preliminary proof of functional relevance; however, the strength of evidence varies by complication. In the diabetic kidney, there was HSP47 upregulation likely involved in renal fibrosis, but expression of cytoprotective HSP27, HSPC/HSP90, HSPA/HSP70, and HSPD1/HSP60 was unchanged in the renal cortex of animals with type 1 diabetes and both HSPC/HSP90 and HSPD1/HSP60 were even downregulated in other models of diabetes. This indicates an overall insufficiency of the compensatory HSP response to diabetes-induced stresses within the kidney. Consistent with this notion, enhanced HSP transcription by inhibiting HSPC/HSP90 was beneficial. In addition, phosphorylated HSPB1 and eHSPA/eHSP70 were enhanced in podocytes and tubular cells, respectively, and both these changes are potentially deleterious as phosphorylated HSPB1 may favour proteinuria and eHSPA/eHSP70 promotes tubule-interstitial inflammation. Therefore, in the kidney the HSP machinery fails to confer protection and some changes may even be detrimental. Similarly, in the retina, diabetes does not evoke the expected HSP response as total HSPB1 was unchanged, HSPD1/HSP60 was reduced in the mitochondria, HSPC/HSP90 was downregulated and only HSPA/HSP70 underwent upregulation. However, there are no intervention studies proving functional relevance with the exception of a protective effect of HSPA/HSP90 inhibition through downregulation of angiogenic factors involved in PDR.

More convincing evidence for a functional role of HSP is available in DSP. Although HSP expression measurement only showed an upregulation of HSPB1 and HSPD1/HSP60, intervention studies proved amelioration of DSP in experimental animals either overexpressing HSPB1 or treated with HSPC/HSP90 to induce HSPA/HSP70. Therefore, boosting the HSP response results in neuroprotection, strongly suggesting that HSP expression in the diabetic neurons is inadequate to counteract cellular stresses.

At variance with microvascular complications, most HSP (HSPC/HSP90, HSPA/HSP70, HSPD1/HSP60) were upregulated in macrovascular disease with the exception of both intra and extracellular HSPB1 that underwent downregulation. However, data were predominantly obtained in non-diabetic atherosclerotic mice and it would be important to obtain data in diabetic animals to clarify if diabetes itself is responsible of the failure to induce a normal stress response. However, both HSPB1 overexpression and HSPC/HSP90 inhibition ameliorated the phenotype of the atherosclerotic plaques in diabetic animals, confirming the presence of a relative deficiency in HSPs in CVD. Data on diabetic wounds are scarce; however, available results indicate that insufficient HSP47 may interfere with wound repair, while HSP90-α accelerates healing.

## 5. Limits of Current Research and Future Perspective

Research on HSPs in the field of diabetic complications has important limitations. Changes in HSP expression in target organs of DM complications are difficult to interpret as a rise in HSP expression may suggest a direct involvement of HSPs in the pathogenic process, a compensatory cytoprotective response or even both in different stages of the disease. For instance, HSPB1 expression was found enhanced in diabetic DRG and this may suggest either a compensatory rise to protect neurons or a mechanism of diabetes-induced neuron injury. Intervention studies have clarified that HSPB1 is protective, but its induction is insufficient, as greater HSPB1 levels are required to confer neuroprotection. Moreover, intra and extracellular HSPs often have opposing effects and studies assessing HSP protein expression cannot discriminate between the two, making difficult to draw any conclusion. For example, intracellular HSPA/HSP70 has cytoprotective effects that are important in defending renal cells exposed to a diabetic milieu, while eHSPA/eHSP70 causes tubule-interstitial damage. These opposing effects of intra/extracellular HSPs may also provide a possible explanation for the highly conflicting results on expression of HSPs in diabetic complications. Therefore, intervention studies targeting specifically either intra or extracellular HSPs are required to gain a deeper insight on the functional role of HSP in DM complications.

HSPs can also be released outside cells in extracellular vesicles (EVs) that are an important new mechanism of cell-to-cell communication. EVs are enriched in HSPA/HSP70, though other HSPs, including HSPB1 and HSPC/HSP90, have also been found. Delivery of HSPs via EVs is very effective as HSPs are present in high concentrations in EVs and they can synergize with other factors enclosed within EVs [153]. For instance, activation of macrophages by HSPA/HSP70 carried by EVs was more than 250-fold higher than the same concentration of the HSP70 in solution [154]. The role of HSPs carried by EVs has been explored mainly in cancer and immunology. However, there is preliminary evidence of relevance in diabetes and associated complications. For example, HSP20 has been recently shown to improve diabetic cardiomyopathy by increasing the release of EVs enriched in HSP20 and other cardioprotective factors [155]. Moreover, fibrocytes-derived exosomes, enriched in HSP90α, accelerated wound closure in diabetic mice in vivo [156]. This is emerging area of research that is expected to grow exponentially in the near future.

It is increasing recognised that HSPs act in a network; therefore, abnormalities of clusters of HSPs are of great relevance. Consistent with this view, repression of ATP-dependent and induction of ATP-independent HSPs has been recently demonstrated in both aging and neurodegenerative diseases [157]. This aspect requires consideration in future research in the diabetes field as it would be important to move from detection of abnormalities in the expression of individual HSPs to the identification of clusters of changes in the chaperome that are of functional relevance and may contribute to insufficient proteostasis.

There is great interest on the potential use of HSPs as therapeutic targets. Because DM causes accumulation of damaged proteins, oxidative stress, altered mitochondrial bioenergetics, and apoptosis, boosting the HSP cytoprotective machinery appears an ideal strategy to prevent and/or reduce the progression of DM complications and available data in experimental animals support this hypothesis. Moreover, the recent development in cancer research of compounds that can rise/lower HSP levels in a specific and safe manner has open the way to pharmacological intervention studies targeting HSPs also in others pathological conditions, including DM-related complications (Table 2). In this respect, newly developed HSPC/HSP90 inhibitors appear very promising. Moreover, Fv-HSP70, a fusion molecule of HSPA/HSP70 to the single-chain Fv fragment of a cell-penetrating antibody, can be used for the intracellular delivery of HSPA/HSP70 and has been recently proven safe in humans [158]. However, given the multitude of HSP functions, their pharmacological modulation may cause undesired effects; therefore, development of compounds targeting specific HSP-client interactions and/or HSP functions would be crucial in pathological conditions requiring long-term therapy as DM complications. Tissue-targeting strategies may also be valuable as pharmacological modulation of HSPs can have opposing effects in different vascular beds of DM complications.

There is an increasing need to identify novel biomarkers of DM complications particularly in view of potential applications in precision medicine. Available data suggest that circulating both HSP and anti-HSP levels may be exploited as biomarkers of diabetic vascular diseases (Table 3); however, studies performed in this area have important limits. They are often conducted on small groups of patients and without adjustment for conventional risk factors and confounders. Sometimes patients with diabetic complications are compared with healthy subjects, not taking into account that DM itself can affect HSP circulating levels. Finally, there are insufficient prospective studies assessing the importance of HSPs/anti-HSPs in predicting outcomes and guiding intervention. Therefore, it would important in the future not only to identify independent associations between HSPs/anti-HSPs and DM complications in large cohorts of well-characterised patients with DM, but also to prospectively validate promising biomarkers for future applications in clinical practise.

## Figures and Tables

**Table 1:** HSP Proteins Subfamilies—Classifications.

HSP Names	Old Names
HSPB	
HSPB1	HSP27; CMT2F; HMN2B; HSP28; HSP25; HS.76067; DKFZp586P1322
HSPB2	HSP27; MKBP; Hs.78846; LOH11CR1K; MGC133245
HSPD1	HSP60; GroEL
HSPA	
HSPA1A	HSP70-1; HSP72; HSPA1
HSPA1B	HSP70-2
HSPA2	Heat-shock 70kD protein-2
HSPA5	BIP; GRP78; MIF2
HSPA8	HSC70; HSC71; HSP71; HSP73
HSPA9	GRP75; HSPA9B; MOT; MOT2; PBP74; mot-2
HSPC	
HSPC1	HSP90AA1; HSPN; LAP2; HSP86; HSPC1; HSPCA; HSP89; HSP90; HSP90A; HSP90N; HSPCAL1; HSPCAL4; FLJ31884
HSPC2	HSP90AA2; HSPCA; HSPCAL3; HSP90
HSPC3	HSP90AB1; HSPC2; HSPCB; D6S182; HSP90B; FLJ26984; HSP90
HSPC4	HSP90B1; ECGP; GP96; TRA1; GRP94; endoplasmin
HSPC5	TRAP1; HSP75; HSP90L

**Table 2:** Summary of Intervention Studies Targeting HSPs in Animal Models of Diabetic Complications.

HSP	Action	Strategy	Specificity	Complication	Animal Model	Effects	Reference
HSP27	Inhibition of phosphorylation	Genetic deletion of MK-2	+/−	Diabetic nephropathy	STZ-induced diabetic MK-2^−/−^ mice	None	[29]
	Inhibition of phosphorylation	PHA666859 (p38 inhibitor)	-	Diabetic retinopathy	STZ-induced diabetic rats	Amelioration of retinal vascular injury	[38]
	Induction	Genetic overexpression	+	Diabetic neuropathy	STZ-induced diabetic hHSP27 transgenic mice	Amelioration of DSP	[52]
HSP90	Inhibition	17-DMAG (HSP90 inhibitor)	+	Diabetic nephropathy	db/db mice HFD	Reduced kidney damage	[78]
	Inhibition	17-DMAG (HSP90 inhibitor)	+	Diabetic nephropathy	STZ-induced diabetic ApoE^−/−^ mice	Reduced albuminuria and mesangial expansion	[80]
	Inhibition	SH-1242/SH-1280 (HSP90 inhibitors)	+	Diabetic retinopathy	STZ-induced diabetic mice	Reduced retinal vascular leakage	[88]
	Inhibition	KU-32 (C-terminal HSP90 inhibitor)	+	Diabetic neuropathy	STZ-induced diabetic mice	Amelioration of DSP	[90]
	Inhibition	17-DMAG (HSP90 inhibitor)	+	Diabetic macrovascular disease	STZ-induced diabetic ApoE^−/−^ mice	Reduced number of atherosclerotic lesions and more stable plaques	[80]
	Inhibition	17-DMAG (HSP90 inhibitor)	+	Diabetic macrovascular disease	STZ-induced diabetic mice	Reduced lesion size and inflammation	[102]
HSP70	Inhibition	PFTμ/ VER (intracellular HSP70 inhibitors)	+	Diabetic nephropathy	STZ-induced diabetic mice	Reduced albuminuria, tubular injury	[111]
	Inhibition	KNK437 (HSF-1 inhibitor)	+/−	Diabetic nephropathy	STZ-induced diabetic mice	Reduced albuminuria tubular injury	[111]
	Inhibition	HSP70 neutralizing Ab (blockade of eHSP70)	+	Diabetic nephropathy	STZ-induced diabetic mice	Reduce albuminuria	[111]

MK-2: MAP kinase-activated protein kinase 2; STZ: streptozotocin; DSP: distal symmetric polyneuropathy; 17-DMAG: 17-dimethylaminoethylamino-17-demethoxygeldanamycin; eHSP70: extracellular HSP70. HSP27 = HSPB1; HSP90 = HSPC; HSP70 = HSPA.

**Table 3:** Summary of Clinical Studies on Circulating HSP and anti-HSP Levels in Patients with Diabetic Complications.

Biomarker	Study Design	Study Population	N	Results	Adjustments	Reference
HSP27	Hospital-based case-control study	DM2 with microvascular complications vs. C	C = 247 DM2 = 195 (DR = 123, DN = 80, DNu = 109)	HSP27 higher in DM2-DN vs. other groups	Gender, age, BMI	[33]
	Nested case-control study EURODIAB PCS	DM1 with and without complications	Controls = 168 Cases = 363	Direct, independent association with DSP OR 2.41 (1.11–5.24)	Conventional risk factors, markers of inflammation, AER	[54]
	Case-control study	Subjects with NGT, IGT and DM2	NGT = 39 IGT = 29 DM2 = 51	Inverse association with nerve function OR 2.51 (1.25, 5.05)	Age, sex, statin, HbA1c	[56]
Anti-HSP27	Nested case-control study EURODIAB PCS	DM1 with and without complications	Controls = 168 Cases = 363	No association with DM1 complications	Age, DM duration, hypertension, HbA1C, smoking, TNF-α	[74]
Urinary HSP70	Case-control study	DM2 (Normo, Micro Macro) vs. C	C = 15 DM2 = 45 (Normo = 15 Micro = 15, Macro = 15)	Urinary HSP70 higher in Micro/Macro than in Normo DM2	None	[112]
HSP70	Case-control study	DM2 with and without albuminuria	DM2-Normo = 40 DM2-Alb = 40	HSP70 higher in Alb than in Normo DM2	None	[113]
	Case-control study	DM2 with and without DR vs. C	C = 70 DM2 without DR = 50 DM2 with DR = 50	HSP70 higher in DM2 with DR	None	[116]
Anti-HSP70	Nested case-control study EURODIAB PCS	DM1 with and without complications	Controls = 168 Cases = 363	Independent, inverse association with DR [OR 0.35 (0.15–0.80)] and CVD [OR 0.39 (0.17–0.87)]	Age, DM duration, hypertension, HbA1c, smoking, TNFα, homocysteine, AER	[117]
HSP60	Cross-sectional UDACS Study	DM patients with and without CVD	DM1 = 147 DM2 = 708 DM without CVD = 607 DM with CVD = 241	HSP60 detectable more frequently in subjects with CVD and MI	Age, sex, ethnic group, smoking	[151]
Anti-HSP60	Nested case-control study EURODIAB PCS	DM1 with and without complications	Controls = 168 Cases = 363	No associations	-	[117]

C: healthy controls, DM1: type 1 diabetes, DM2: type 2 diabetes, NGT: normal glucose tolerance, IGT: impaired glucose tolerance, Normo: normoalbuminuria, Micro: microalbuminuria; Macro: macroalbuminuria; Alb: albuminuria; DR: diabetic retinopathy; DN: diabetic nephropathy; DNu: diabetic neuropathy; CVD: cardiovascular diseases; DSP: distal symmetric polyneuropathy; MI: myocardial infarction; AER: albumin excretion rate, TNF-α: tumor necrosis factor. N: number.
